# A case report of long-term asymptomatic primary hypothyroidism treated with levothyroxine and dexamethasone

**DOI:** 10.15190/d.2024.5

**Published:** 2024-06-30

**Authors:** Kinal Paresh Bhatt, Larri Rudman, Daniela Ramos Padilla, Kamal Akbar, Nicole Clarke, Paulraj Rahulraj, George Michel

**Affiliations:** ^1^Larkin Community Hospital, South Miami, FL, USA

**Keywords:** Primary hypothyroidism, hypertrophic thyroiditis, levothyroxine, dexamethasone.

## Abstract

Hypothyroidism is an underactive thyroid gland that is diagnosed based on the laboratory findings. The risk is higher in women over the age of 60, pregnancy, patients with a prior history of head and neck irradiation, patients with autoimmune disorders and/or type 1 diabetes, family history, positive thyroid peroxidase antibodies, and medication adverse effects. The primary screening test for thyroid dysfunction is serum thyroid stimulating hormone (TSH) testing. Abnormal findings will require a follow-up testing of serum thyroxine (T4). Abnormally high TSH and low T4 will confirm the diagnosis of hypothyroidism, also known as “overt” hypothyroidism. No consensus exists on the treatment threshold or better clinical outcome for hypothyroidism. Generally, a TSH level greater than 10.0 mIU/L is considered optimal for treatment initiation for symptomatic and asymptomatic hypothyroid patients. The present case emphasizes the importance of close observation in a patient with primary hypothyroidism findings and the importance of adequate treatment. When treated with thyroxine replacement, both autoimmune and nonautoimmune mechanisms of primary hypothyroidism may contribute to iatrogenic thyrotoxicosis. Levothyroxine has a very narrow therapeutic index; therefore, to avoid adverse effects of levothyroxine-induced iatrogenic thyrotoxicosis, dexamethasone was added as an adjunct medication. Dexamethasone inhibits TSH, further reducing the release of T3 and T4 from the anterior pituitary gland. We advised the patient to have an outpatient follow-up for appropriate follow-up and educated him about the importance of continuity of care for his diagnosis.

## INTRODUCTION

Hypothyroidism is an underactive thyroid gland that is diagnosed based on the laboratory findings.Although thyroid gland abnormalities are among the most common endocrine diagnoses, the United States Preventive Services Task Force (USPSTF) has concluded that insufficient evidence exists to screen asymptomatic adults and nonpregnant patients routinely. However, the American Thyroid Association recommends screening for high-risk patients should begin at the age of 35 and every five years after that. High-risk patients include women over the age of 60, pregnancy, patients with a prior history of head and neck irradiation, patients with autoimmune disorders and/or type 1 diabetes, family history, positive thyroid peroxidase antibodies, and medication adverse effects. The primary screening test for thyroid dysfunction is serum thyroid stimulating hormone (TSH) testing. Abnormal findings will require a follow-up testing of serum thyroxine (T4). Abnormally high TSH and low T4 will confirm the diagnosis of hypothyroidism, also known as “overt” hypothyroidism^1,2,3^ (Table 1).

**Table 1:** Thyroid function test interpretation

Condition	TSH	Free T4	Free T3
Primary hypothyroidism	High	Low	Low
Subclinical hypothyroidism	High	Normal	Normal
Central hypothyroidism	Low	Low	Low

As per the National Institute of Health (NIH), 5 out of 100 Americans over 12 years of age have hypothyroidism, most commonly in those above 60. Women are more likely to develop hypothyroidism than men. Because it is a slowly progressive disease, the patient may not experience symptoms for months or even years. Common symptoms of hypothyroidism are fatigue, weight gain, cold intolerance, joint and muscle pain, dry skin, hair loss, heavy or irregular menstrual periods, constipation, fertility problems, bradycardia, and depression. All patients with symptoms of hypothyroidism should be evaluated for hypothyroidism. Both overt and subclinical hypothyroidism patients may be asymptomatic. Compared to high TSH and low T4 in overt hypothyroidism, the subclinical hypothyroidism patient’s lab will show high TSH and normal T4. The spectrum of hypothyroidism is asymptomatic subclinical hypothyroidism, hyperthyroidism to asymptomatic overt hypothyroidism, and hyperthyroidism to symptomatic overt hypothyroidism^2,3,4,5^.

No consensus exists on the treatment threshold or better clinical outcome for hypothyroidism. Generally, a TSH level greater than 10.0 mIU/L is considered optimal for treatment initiation for symptomatic and asymptomatic hypothyroid patients. Overdiagnosis and overtreatment likely result from thyroid dysfunction screening, mainly because the diagnosis often relies on laboratory values rather than clinical symptoms that are not always consistent or reliable. Patients may require repeat laboratory values as a basis for diagnosis or to determine treatment strategy, as many patients revert to normal thyroid function without any treatment. The overt and subclinical thyroid can be treated with synthetic T4, a prohormone with a half-life of 7 days, allowing one daily treatment to result in constant T4 and T3 concentrations. The treatment aims to keep serum TSH within the 0.5 to 5.0 mU/L reference range. This range is slightly higher at approximately 7.5 mU/L for older patients (≥70 years)^2,6,7,8^.

## CASE PRESENTATION

A previously healthy, seemingly asymptomatic 49-year-old male, a cruise line worker with a past medical history of hypertension, was brought to the Larkin Community Hospital (LCH) Emergency Room (ER) after he suffered a crush injury to his right hand while fixing an engine at work. He sustained lacerations to his right thumb, correct 4th and 5th digits that were approximated with silk sutures by the cruise doctor. Plastic and reconstructive surgery (PRS) teams were consulted to evaluate the patient's wounds further. The 4th digit nail bed repair was done at the bedside by the PRS team, and the patient was scheduled for right 4th and fifth digit exploration, complex soft tissue rearrangement, and possible skin substitute placement with a skin graft.

**Table 2:** Laboratory evaluation throughout hospitalization

	Day 0	Day 1	Day 2	Day 3	Day 4	Day 5	Day 6
T3 (mlU/L)	0.4	–	–	0.8	–	–	–
T4FREE (mlU/L)	0.07	–	–	1.13	–	–	–
T4 (mlU/L)	–	–	–	–	8	10.9	–
TSH (mlU/L)	153 & 172	–	–	–	–	–	–
ESR (mm/hr)	23	–	–	–	–	–	–
AST (U/L)	90	62	56	58	52	46	60
CHOL (mmol/L)	–	–	310	–	–	–	–
TRIG (mmol/L)	–	–	168	–	–	–	–
ALT (U/L)	48	33	31	35	31	26	53
HDL (mmol/L)	–	–	59	–	–	–	–
ALKP (U/L)	45	42	45	44	45	40	75
LDL (mmol/L)	–	–	217	–	–	–	–
CK (U/L)	2797	1326	–	–	–	–	297
THYROYDA	-	-	16	-	-	-	-
TPOAB	-	-	16	-	-	-	-

As part of his pre-op workup, the patient had a series of laboratory tests performed. The emergency room (ER) referred the patient to the Internal Medicine (IM) team because of a high TSH level of 153 mlU/L. His repeat TSH was 172 with a low T3 of 0.4 and a low T4 of 0.07, confirming the diagnosis of primary hypothyroidism. The patient denied any family history of thyroid disorders or autoimmune diseases. Only laboratory findings suggestive of hypothyroidism included initially elevated liver function tests (LFTs) (alanine transaminase (ALT): 90, aspartate transaminase (AST): 62); hyponatremia (132); mixed dyslipidemia (total cholesterol (TC): 310, triglycerides (TG): 168, low-density lipoprotein (LDL): 217, high-density lipoprotein (HDL): 59). He also had elevated creatine kinase (CK) (2797), possibly due to the crush injury, however such can also be the case owing to decreased renal clearance (Table 2). The patient also did not present with the typical signs and symptoms of hypothyroidism (including but not limited to fatigue, depressed mood, inexplicable weight gain, decreased appetite, hair loss, dry skin, periorbital puffiness, constipation, etc.).

**Figure 1:**
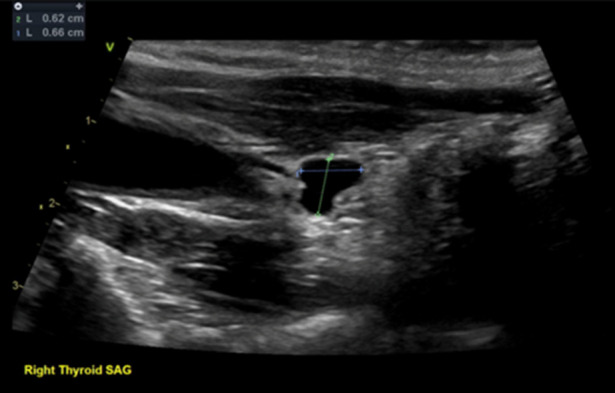
Ultrasound image of patient’s thyroid gland

The patient denied any difficulty in swallowing or any shortness of breath. There was no history of chronic medical conditions except hypertension, no past surgical history, or regular medication use. Neck examination showed no scars or abnormal thyroid movement with deglutition, no thyroid nodules, or palpable lymph nodes noted. Lower limb tone and power were intact despite slow relaxation of ankle reflexes. Subsequently, anti-thyroid peroxidase (anti-TPO) antibodies at 16 and TSH receptor antibodies (TRAb) at 16 were obtained; although titers seem small, this may suggest long-standing Hashimoto's hypothyroidism; levels are not increased, likely due to atrophy of the gland (which is a phenomenon that can be seen in long-standing disease states). A thyroid ultrasound demonstrated a very atrophic thyroid gland with a benign cyst replacing his right thyroid lobe (Figure 1).

The patient was diagnosed with severe primary hypothyroidism and started on levothyroxine 280 mcg intravenous push (IVP), dexamethasone 4 mg IVP, losartan (Cozaar) 25 mg PO QD, and NS 0.9% 100 ml/hr, and the IM team consulted the endocrine team. If Free T4 is detectable at any point, the endocrine team would consider transitioning him to an oral dose. The patient should be on levothyroxine (Synthroid) 100mcg PO daily based on his weight. Although the patient had no symptoms at this time, it is recommended to be discharged with some level of detectable Free T4 in the blood.

Once that is done, we can consider transitioning to levothyroxine (Synthroid) 100mcg PO daily and discharge with said dose on a branded levothyroxine replacement. The patient remains asymptomatic. He was treated with one dose of dexamethasone 4mg IVP and three doses of Levothyroxine sodium 270mcg IVP over three consecutive days. The patient had his T4 levels checked. The levels normalized after he was given the three doses of levothyroxine (Synthroid). So, the patient was discharged with levothyroxine 100 mcg PO daily. He is advised to follow up with outpatient PCP within two weeks upon discharge.

## DISCUSSION

Thyroid hormone plays a crucial role in lipid and energy metabolism. This is why, irrespective of the cause, thyroid hormone can have clinical implications on nearly all organs. The insidious onset and late presentation of symptoms make hypothyroidism difficult to diagnose. Our patient was originally from the Philippines, where the prevalence of hypothyroidism is considered low (8.53%). Iodine deficiency is the most common cause of hypothyroidism globally; however, the Philippines is a repleted country. Compared to autoimmune causes, such as Hashimoto’s in the United States, subclinical hypothyroidism is the most common cause of hypothyroidism in the Philippines, ten times more common in older women than men^9,10,11^. This patient did not present with the typical signs and symptoms of hypothyroidism, including but not limited to fatigue, depressed mood, inexplicable weight gain, decreased appetite, hair loss, dry skin, periorbital puffiness, constipation, difficulty swallowing, shortness of breath, etc. The patient also denied prior history of head and neck irradiation, personal or family history of autoimmune disorders and/or type 1 diabetes, or past surgical history. He has only taken anti-hypertensive medication (valsartan 25 mg po daily) without any side effects over the years. The patient reported he has always felt healthy and denied knowledge of thyroid diagnosis.A thyroid ultrasound demonstrated a very atrophic thyroid gland with a benign cyst replacing his right thyroid lobe. This is commonly known as atrophic thyroiditis and is considered an extreme form of primary hypothyroidism. Chronic autoimmune thyroiditis (Hashimoto’s) is the most common cause of primary hypothyroidism in iodine-repleted countries. Generally, most cases of Hashimoto’s have diffusely large goiters, which was not observed in this patient. Autoimmune hypothyroidism with small thyroid glands is referred to as Ord’s hypothyroidism.In Hashimoto’s and Ord’s thyroiditis, thyroid volume follows a normal volume distribution. Antibodies to thyroid peroxidase and thyroglobulin are thought to destroy follicles by invasion of the T-lymphocytes. This patient had positive anti-TPOAb and anti-TRAb, further suggesting an autoimmune cause^10,12,13,14^.Both hyper- and hypothyroidism can cause hypertension through different mechanisms. In hyperthyroidism, increased heart rate leads to increased cardiac output. An increased level of T3 increases cardiac contractility as well. The high levels of atrial natriuretic peptide, brain natriuretic peptide, endothelin-1, and adrenomedulin may also contribute to hypertension in hyperthyroidism patients. Hypertension caused by hyperthyroidism increases mortality due to cardiovascular origins by 20% ^15^. In hypothyroidism, the heart muscle is weakened and, over time, becomes less efficient. Bradycardia causes decreased ventricular filling and cardiac contractility, leading to low cardiac output. This, in turn, causes increased systemic vascular resistance, leading to hypertension. In hypertension caused by thyroid disorders, anti-hypertensive medications alone may not help control the blood pressure (BP). Decreased metabolic activity of hypothyroidism leads to a decline in peripheral oxygen demand and reduced renin clearance, leading to expansion of blood volume through sodium reabsorption. This mechanism rarely leads to heart failure^15^. This patient’s BP on admission was 155/90, and heart rate (HR) of 66. This high reading also may be secondary to blood loss upon injury. However, the patient admitted he was frequently told by his primary care physician he might need adjunctive anti-hypertensive medication if his BP is not controlled on the current regimen of valsartan 25 mg PO daily. Thyroid hormones increase expression of HMG-CoA reductase enzyme in the liver, leading to increased cholesterol synthesis. Hence, hypothyroidism should lead to decreased hepatic cholesterol synthesis. However, T3-mediated effects on the sterol regulatory element-binding protein-2 (SREBP-2) decreases cell-surface LDL-cholesterol receptors. This in turn, leads to reduced plasma LDL-cholesterol clearance and increased apo-B lipoproteins. These multifactorial and complex mechanism leads to increased total cholesterol, LDL, HDL and TG levels, which ultimately increases risk of non-alcoholic fatty liver disease. Several observational studies have shown levothyroxine treatment may alter lipid metabolism, except the patient with underlying hyperlipidemia^16^. Our patient had high LDL (217), TC (310) and TG (168) on admission. However, he had normal liver function tests and no signs and symptoms of jaundice or other liver pathologies. The patient was started on atorvastatin 20 mg PO daily.

**Figure 2:**
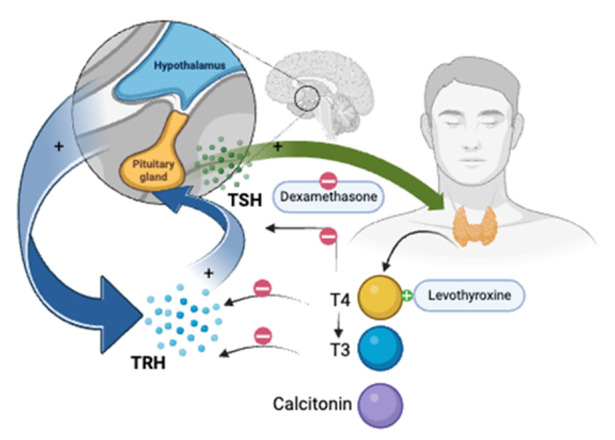
Mechanism of action of levothyroxine and dexamethasone

On day 0, upon laboratory findings, he was treated with levothyroxine sodium 280 mcg intravenously (IV) and dexamethasone 4 mg IV. He received two more doses of levothyroxine on Day 1 and Day 2 of his hospitalization. On Day 4, to further increase thyroid binding globulin (TBG) stores, the endocrinology team continued to treat the patient with a new dose of levothyroxine at 400 mcg IV. The endocrine team determined that once the T4 is detectable, the patient would transition to a PO dose of 100 mcg PO daily (based on his weight). On Day 5, new T4 levels were repeated by the endocrine team and measured at 8.0, and the patient was transitioned to levothyroxine 100 mcg po daily. The patient was observed for an additional 24 hours and discharged on Day 6. The patient remained asymptomatic during his hospitalization. Levothyroxine is a synthetic form of T4 with the same physiological effects as human-secreted thyroxine T4. It increases the metabolic rate, decreases the production of TSH from the anterior pituitary gland, and helps maintain thyroxine levels when there is a deficiency. When treated with thyroxine replacement, both autoimmune and nonautoimmune mechanisms of primary hypothyroidism may contribute to iatrogenic thyrotoxicosis. Levothyroxine has a very narrow therapeutic index; therefore, to avoid adverse effects of levothyroxine-induced iatrogenic thyrotoxicosis, dexamethasone was added as an adjunct medication. Dexamethasone inhibits TSH, further reducing the release of T3 and T4 from the anterior pituitary gland^17,18^ (Figure 2).

## CONCLUSION

Both autoimmune and nonautoimmune mechanisms of primary hypothyroidism may contribute to iatrogenic thyrotoxicosis when treated with thyroxine replacement. Levothyroxine has a very narrow therapeutic index, therefore, to avoid adverse effect of levothyroxine induced iatrogenic thyrotoxicosis, dexamethasone was added as an adjunct medication. Dexamethasone has an inhibitor effect on TSH, which further reduces release of T3 and T4 from anterior pituitary gland. We advised the patient to have an outpatient follow up for appropriate follow up and educated the patient about importance of continuity of care for his diagnosis. This patient did not present with the typical signs and symptoms of hypothyroidism. A thorough blood test prior to any major or minor surgical procedure may significantly help reduce the risk of surgical complications.
